# Beta cells deficient for *Renalase* counteract autoimmunity by shaping natural killer cell activity

**DOI:** 10.3389/fimmu.2024.1403752

**Published:** 2024-06-04

**Authors:** Kevin Bode, Siying Wei, Isabella Gruber, Jian Li, Stephan Kissler, Peng Yi

**Affiliations:** ^1^ Section for Immunobiology, Joslin Diabetes Center, Boston, MA, United States; ^2^ Section for Islet Cell and Regenerative Biology, Joslin Diabetes Center, Boston, MA, United States; ^3^ Department of Medicine, Harvard Medical School, Boston, MA, United States; ^4^ Diabetes Program, Harvard Stem Cell Institute, Cambridge, MA, United States

**Keywords:** autoimmunity, type 1 diabetes, transplantation, beta cell, NK cell, CD47, Ceacam1, Tgfβ1

## Abstract

Type 1 diabetes (T1D) arises from autoimmune-mediated destruction of insulin-producing pancreatic beta cells. Recent advancements in the technology of generating pancreatic beta cells from human pluripotent stem cells (SC-beta cells) have facilitated the exploration of cell replacement therapies for treating T1D. However, the persistent threat of autoimmunity poses a significant challenge to the survival of transplanted SC-beta cells. Genetic engineering is a promising approach to enhance immune resistance of beta cells as we previously showed by inactivating the *Renalase* (*Rnls*) gene. Here, we demonstrate that *Rnls* loss of function in beta cells shapes autoimmunity by mediating a regulatory natural killer (NK) cell phenotype important for the induction of tolerogenic antigen-presenting cells. *Rnls*-deficient beta cells mediate cell**–**cell contact-independent induction of hallmark anti-inflammatory cytokine Tgfβ1 in NK cells. In addition, surface expression of regulatory NK immune checkpoints CD47 and Ceacam1 is markedly elevated on beta cells deficient for *Rnls*. Altered glucose metabolism in *Rnls* mutant beta cells is involved in the upregulation of CD47 surface expression. These findings are crucial to better understand how genetically engineered beta cells shape autoimmunity, giving valuable insights for future therapeutic advancements to treat and cure T1D.

## Graphical Abstract

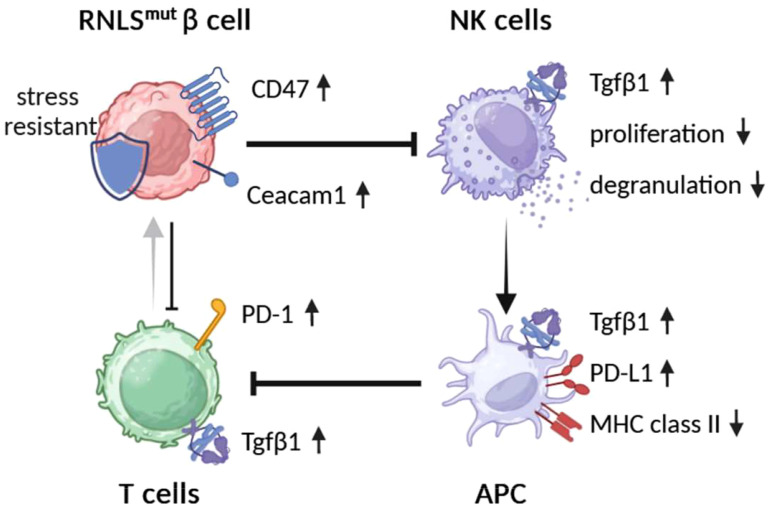

## Introduction

In type 1 diabetes (T1D), the autoimmune process leads to the selective destruction of insulin-producing beta cells within pancreatic islets. Upon extensive elimination of the majority of beta cell mass by autoimmunity, a curative strategy for T1D requires the replenishment of functional beta cell mass to completely restore the patient’s capacity for insulin production (1). The latest progress in the manufacturing of beta cells derived from stem cells (SCs) has rendered beta cell replacement therapy a viable promise (2). Despite these advancements, a significant challenge in translating this strategy to clinical application lies in our current inability to protect beta cells against recurrent autoimmune attacks without resorting to broad immunosuppressive treatments. Exacerbating this obstacle is the existing limitation in generating patient-specific SC-derived beta cells, underscoring the need to address both autoimmunity and alloimmune responses in any cellular therapy for T1D in the foreseeable future (3). To address these challenges, various research groups have started to genetically engineer SC-derived beta cells for enhanced resilience against immune-mediated destruction. Notably, most efforts focus on the deletion of genes encoding major histocompatibility complex (MHC) molecules mandatory for antigen presentation and activation of T cells, coupled with the incorporation of immune inhibitory ligands such as CD47 to prevent stimulation of innate immune cells (4–10).

In our pursuit of discovering novel immune-regulatory targets to protect beta cells from autoimmune destruction, we previously performed an unbiased genome-wide *in vivo* CRISPR screen, and found that *Renalase* (*Rnls*) deletion is able to protect beta cells from stress-induced cell death and autoimmunity (9). Beta cells lacking *Rnls* not only exhibit enhanced resilience to stress but also undergo comprehensive metabolic alterations favoring glucose metabolism (9, 10). Moreover, we have shown previously that *Rnls*-deficient beta cells orchestrate localized shifts in the overall immune cell composition within the graft, primarily characterized by the enhanced infiltration of CD4^+^ T cells, reduced numbers of natural killer (NK) cells, and the accumulation of tolerogenic antigen-presenting cells (APCs) defined by diminished MHC class II expression coupled with elevated levels of programmed cell death 1 ligand 1 (PD-L1). We also demonstrated that the survival advantage of beta cells lacking *Rnls* is attributed to the induced expression of PD-L1 on APCs. Beta cells deficient for *Rnls* promote a significant transcriptional change in CD45^+^ immune cells within the graft towards hallmark anti-inflammatory genes such as transforming growth factor beta 1 (Tgfβ1) (10). However, the immune cell population responsible for initiating the immune-regulatory cross-talk to reduce autoimmunity against *Rnls*-deficient beta cell grafts remained elusive.

As Tgfβ1 is a known inducer of PD-L1 upregulation on APCs in certain tumors as well as in pancreatic islet transplanation (11), and NK cells harbor a significant source of Tgfβ1 production (12), we have now investigated if graft-infiltrating NK cells play an important role for mediating the immuno-protective regulation of *Rnls* mutant beta cells. NK cells are not solely responsible for killing transformed or stressed target cells, but are also crucial to modulate the activation and phenotype of immune cells such as APCs (13). Here, we show that depletion of NKp46^+^ innate lympoid cells in an experimental model of beta cell transplantation abrogated the induction of tolerogenic APCs consequently preventing the survival advantage of beta cell grafts deficient for *Rnls*. Using *in vitro* co-culture systems, we demonstrated that beta cells lacking *Rnls* are potent modulators of NK cell activation shown for both mouse-derived NIT-1 beta cells and human SC-derived beta-like cells. Elevated cell surface expression of key NK cell inhibitory molecules on beta cells deficient for *Rnls* such as Cluster of differentiation 47 (CD47) and Carcinoembryonic antigen-related cell adhesion molecule 1 (Ceacam1) are likely to play a major role in suppressing NK cell activation by cell–cell interaction. However, upregulation of Tgfβ1 in NK cells mediated by *Rnls* deletion in beta cells is independent of cell–cell contact demonstrating multifaced propsects of NK regulation.

## Results

### Protective immune-regulation by Rnls^mut^ beta cells requires NKp46^+^innate lymphoid cells

**Figure 1:**
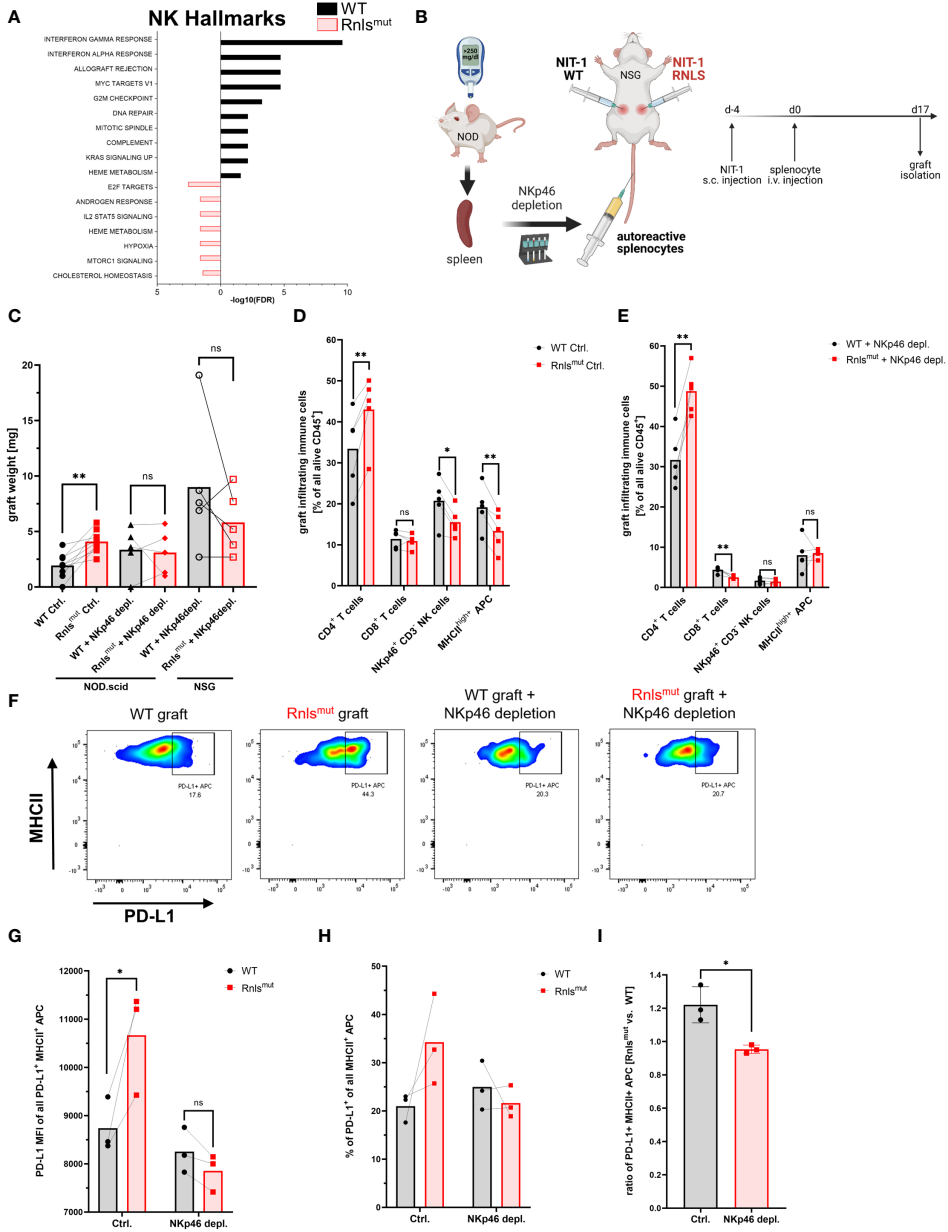
NKp46^+^ innate lymphoid cells are crucial for protective immune-regulation by *Renalase* mutant (Rnls^mut^) NIT-1 beta cells *in vivo.* **(A)** Hallmark gene expression analysis of natural killer (NK) cells derived from indicated NIT-1 beta cell grafts showing the 10 most significantly changed pathways using 213 (Rnls^mut^) or 506 (WT) most significantly upregulated genes (*p* ≤ 0.05) as input. Raw data used in this panel were obtained from experiments performed previously. Datasets from our scRNAseq experiments are available from the Gene Expression Omnibus (https://www.ncbi.nlm.nih.gov/geo/) under accession number GSE226361 as described before (10). **(B)** Schematic representation of the experimental design comparing paired WT and Rnls^mut^ NIT-1 beta cell grafts. Cells were injected s.c. into opposite flanks of immunodeficient mice, followed by i.v. injection of autoreactive splenocytes with or without preceding depletion of NKp46^+^ innate lymphoid cells. Grafts were harvested and scaled, and immune cells were characterized by flow cytometry. **(C)** Weight of paired grafts from mice with or without depletion of NKp46**^+^** cells from autoreactive splenocytes is shown. Results represent the mean of nine (Ctrl. condition) or five (NKp46^+^ depletion) paired biological replicates from two combined independent experiments using NOD.scid or NSG recipient mice as indicated. **(D, E)** Quantification of immune cell subpopulations derived from five paired ctrl. NIT-1 beta cell grafts **(D)** or NKp46^+^ depleted grafts **(E)** as determined by flow cytometry. Results represent the mean of five paired biological replicates. **(F–I)** Representative flow cytometry data **(F)** or quantification **(G–I)** showing indicated expression level of PD-L1 on indicated immune cells derived from paired WT and *Rnls*-deficient grafts as determined by flow cytometry. The ratio of PD-L1^+^ MHCII^+^ cells comparing Rnls^mut^ *vs.* WT NIT-1 cell grafts shown in **(I)** are derived from data shown in **(H)**. Results represent the mean of three paired biological replicates. *p < 0.05, **p < 0.01, ns *p* > 0.05 (paired two-tailed *t*-test). Data obtained from scRNAseq **(A)** and from NKp46^+^ depletion experiments using NSG **(C-E)** or NOD.scid **(C, F–I)** recipient mice are derived from independent experiments. Data of four out of nine mice shown for the Ctrl. condition in **(C)** are partially derived from one previously shown experiment performed in parallel with NKp46 depletion using NOD.scid mice ^10.^ Data of five out of nine mice are derived from one newly performed experiment in parallel with NKp46 depletion using NSG mice.

We previously investigated the consequences of *Rnls* deletion on immune cell infiltration and activation by transplantation of the syngeneic mouse beta cell line NIT-1 into Non-obese diabetic (NOD) mice, a model of T1D. Single-cell RNA sequencing (scRNAseq) of graft-infiltrating CD45^+^ cells revealed that *Rnls* mutant (Rnls^mut^) beta cells broadly influence immune cell activation and metabolism towards anti-inflammatory oncostatin M and Tgfβ1 expression, accompanied by enriched expression of genes important for glycolysis. In addition to elevated numbers of CD4^+^ T cells and tolerogenic PD-L1^+^ APCs, beta cell grafts deficient for *Rnls* showed markedly reduced frequency of NK cells. Because we previously demonstrated that PD-L1 blockade abrogates the advantage of Rnls^mut^ beta cells to survive autoimmunity, and NK cells are known to modulate APC maturation and activation, we now investigated the role of NK cells in protection of Rnls^mut^ beta cells in more detail (10). The gene expression profile of both graft-infiltrating NK cells and closely related type 1 innate lymphoid cells (ILC1) demonstrated markedly increased expression of genes involved in pathways of cellular activation and inflammation (interferon responses, allograft rejection, etc.) when derived from WT beta cell grafts compared to Rnls^mut^ (**Figure 1A** and **Supplementary Figures 1A–C**). Whereas ILC1 showed downregulated gene expression of inflammatory cytokines interferon gamma (Ifng) and tumor necrosis factor alpha (Tnf), NK cells upregulated gene expression of immune-regulatory Tgfβ1 when infiltrated in Rnls^mut^ beta cell grafts (**Supplementary Figures 1A, B**). Interestingly, the frequency of NK cells in Rnls^mut^ beta cell grafts was reduced not only in late stages (10), but also at earlier time points preceding the detection of graft weight difference between control and Rnls^mut^ (**Supplementary Figures 1D, E**; see **Supplementary Figure 2** for gating strategy). Of note, the ILC1 frequency was not changed between the grafts and only represented a vast minority of infiltrating immune cells (≤1%) compared to NK cells (up to 14% according to scRNAseq analysis) (10), indicating that ILC1 likely does not play a major role in mediating the protective immune-regulation caused by Rnls^mut^ beta cells. These observations strengthened our hypothesis that NK cells might be crucial for regulating autoimmunity within Rnls^mut^ grafts. To investigate the importance of NK cells in shaping autoimmunity *in vivo*, we depleted NKp46^+^ innate lymphoid cells from autoreactive splenocytes before adoptive transfer into NIT-1 beta cell graft-bearing mice (**Figure 1B**, **Supplementary Figures 1F–H**). The isogenic WT and Rnls^mut^ beta cells were transplanted subcutaneously (s.c.) on opposite flanks of the same recipient mice following intravenous (i.v.) injection of diabetogenic splenocytes similar to described before (9, 10). Strikingly, NKp46-depleted autoreactive splenocytes lost the ability to protect Rnls^mut^ cells from autoimmunity in both immunodeficient recipients, NOD-*Prkdc^scid^* (NOD.scid) mice and NOD.scid gamma (NSG) mice completely deficient for functional T, B, and NK cells (**Figure 1C**). Whereas the accumulation of CD4^+^ T cells was independent of innate lymphoid cells, the reduced frequency of mature MHCII^high+^ APCs and downregulation of MHCII expression level on APCs in Rnls^mut^ grafts completely depended on the presence of graft-infiltrating NKp46^+^ immune cells (**Figures 1D, E**, **Supplementary Figure 1I**). In the absence of innate lymphoid cells within Rnls^mut^ beta cell grafts, MHCII^high+^ APCs lost the ability to upregulate the immune checkpoint PD-L1, thus forfeiting the survival advantage over WT beta cells as shown before (**Figures 1G, H**) (10). In summary, these observations clearly demonstrated that graft-infiltrating NKp46^+^ innate lymphoid cells are crucial for the induction of a tolerogenic APC phenotype important to reduce autoimmune destruction of Rnls^mut^ beta cells.

### Rnls*^mut^ NIT-1 beta cells shape NK cell activation towards a Tgfβ1^+^ regulatory phenotype*

**Figure 2:**
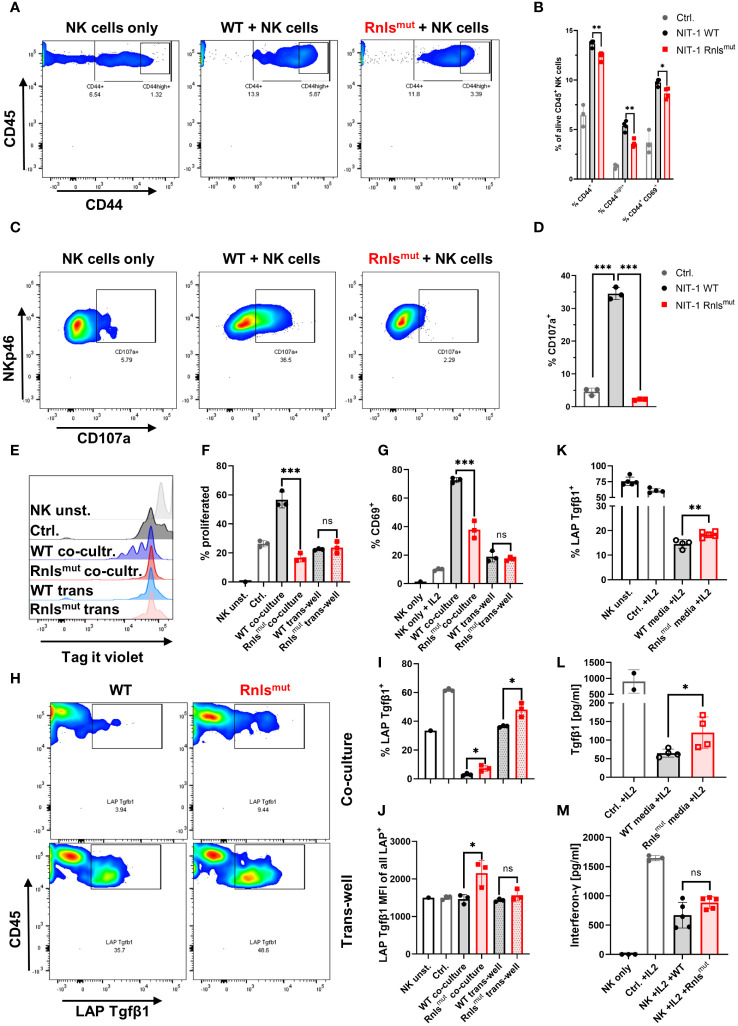
Rnls^mut^ NIT-1 beta cells broadly affect natural killer (NK) cell function *in vitro*. Interleukin 2 (IL2) stimulated primary mouse NK cells were co-cultured with indicated NIT-1 beta cells for 72 h. **(A–D)** Data derived from single-dose IL2 (20 ng/mL)-treated co-culture experiments showing representative flow cytometry plots **(A, C)** or related quantifications **(B, D)** of indicated activation marker **(A, B)** or cytotoxic activity marker CD107a **(C, D)**. **(E-I)** NK cells were repetitively stimulated with IL2 (100 ng/mL) every 24 h together with indicated NIT-1 cells in a co-culture or trans-well setting. **(E, F)** Representative flow cytometry data **(E)** or quantifications **(F)** showing NK cell proliferation of indicated conditions. **(G)** Quantification of surface expression of activation marker CD69 on NK cells. **(H–J)** Representative flow cytometry data **(H)** or quantifications of indicated conditions **(I, J)** showing expression level of membrane-bound latency-associated peptide (LAP)/Tgfβ1 expression on NK cells. **(K, L)** Conditioned media experiments showing LAP/Tgfβ1 surface expression on NK cells **(K)** or secreted amounts of Tgfβ1 derived from NK cells **(L)**. **(M)** Quantification of interferon-γ (Ifng) secretion by NK cells in co-culture with indicated NIT-1 cells. Results represent the mean ± SD from one out of two **(A, B, E–J, L)** or three **(C, D, K, M)** independent experiments (*n* = 3–5). ***p < 0.001, **p < 0.01, *p < 0.05, ns *p* > 0.05, (unpaired, two-tailed *t*-test).

After showing that NKp46^+^ innate lymphoid cells are important to mediate protective immune-regulation leading to prolonged graft survival of Rnls^mut^ beta cells, we subsequently investigated if Rnls^mut^ beta cells directly regulate the activation of innate lymphoid cells. Hence, we purified splenic NKp46^+^ cells (further declared as NK cells as ILC1 only represents approximately 5%–10% of all NKp46^+^ cells in the spleen) (14) by negative selection to collect “untouched” cells for functional *in vitro* co-culture experiments. Activation of NK cells was achieved by supplementation of interleukin-2 (IL2), well-known to enhance the proliferation and effector function of NK cells (15). Co-culture with Rnls^mut^ NIT-1 beta cells significantly reduced NK cell activation indicated by impaired expression of activation marker CD44 and CD69 compared to WT, but did not completely abolished NK cell activation (**Figures 2A, B**). However, the surface expression of degranulation marker CD107a (also known as LAMP1) on NK cells, a molecule that predicts cytotoxic activity (16), was strikingly abolished when co-cultured with Rnls^mut^ NIT-1 beta cells (**Figures 2C, D**). The ability of NK cells to proliferate following repeated IL2 stimulations was also completely prevented when co-cultured with Rnls^mut^ NIT-1 beta cells. This effect was solely dependent on cell–cell contact as separation of NK cells and Rnls^mut^ beta cells in trans-well abrogated the anti-proliferative function of Rnls^mut^ beta cells (**Figures 2E, F**). The cell–cell contact dependency on NK cell regulation by Rnls^mut^ NIT-1 beta cells also became evident for regulation of activation marker CD69 (**Figure 2G**). As described above, differential gene expression analysis of graft-infiltrated NK cells showed enriched Tgfβ1 when derived from Rnls^mut^ NIT-1 beta cell grafts (**Supplementary Figure 1A**). To investigate if Tgfβ1 is also upregulated on protein level, we stained NK cells for latency-associated peptide (LAP) representing membrane-bound Tgfβ1. Indeed, NK cells co-cultured with Rnls^mut^ NIT-1 beta cells demonstrated elevated Tgfβ1 cell surface expression compared to WT NIT-1 beta cells. In contrast to the modulation in NK proliferation, Rnls^mut^ NIT-1 beta cells drive Tgfβ1 expression independent of cell–cell contact (**Figures 2H, I, L**). The fact that Rnls^mut^ NIT-1 beta cells significantly upregulated Tgfβ1 expression and secretion in NK cells by soluble factors was demonstrated by two different methods, in a trans-well assay (**Figures 2H–L**) and by supplementation of conditioned media derived from Rnls^mut^ NIT-1 beta cells in comparison to supernatant of WT cells (**Figures 2K, L**). However, the increased intensity of Tgfβ1 expression on Tgfβ1^+^ cells mediated by Rnls^mut^ NIT-1 beta cells was dependent on cell–cell contact (**Figure 2J**). Next to Tgfβ1, the pro-inflammatory cytokine Ifng is also known to facilitate the upregulation of PD-L1 (11). However, the secretion of Ifng was not significantly affected when NK cells were co-cultured with Rnls^mut^ NIT-1 cells compared to WT (**Figure 2M**). In summary, Rnls^mut^ NIT-1 beta cells directly shape NK cell activity in multiple modes, dependent and independent of cell–cell contact. The presence of NIT-1 beta cells deficient for *Rnls* causes NK cells to fail to proliferate as well as to lose cytotoxic activity in response to IL2, but at the same time elevate the expression level of anti-inflammatory Tgfβ1.

### NIT-1 *beta cells deficient for Rnls upregulate NK immune checkpoint molecules CD47 and Ceacam1*

**Figure 3:**
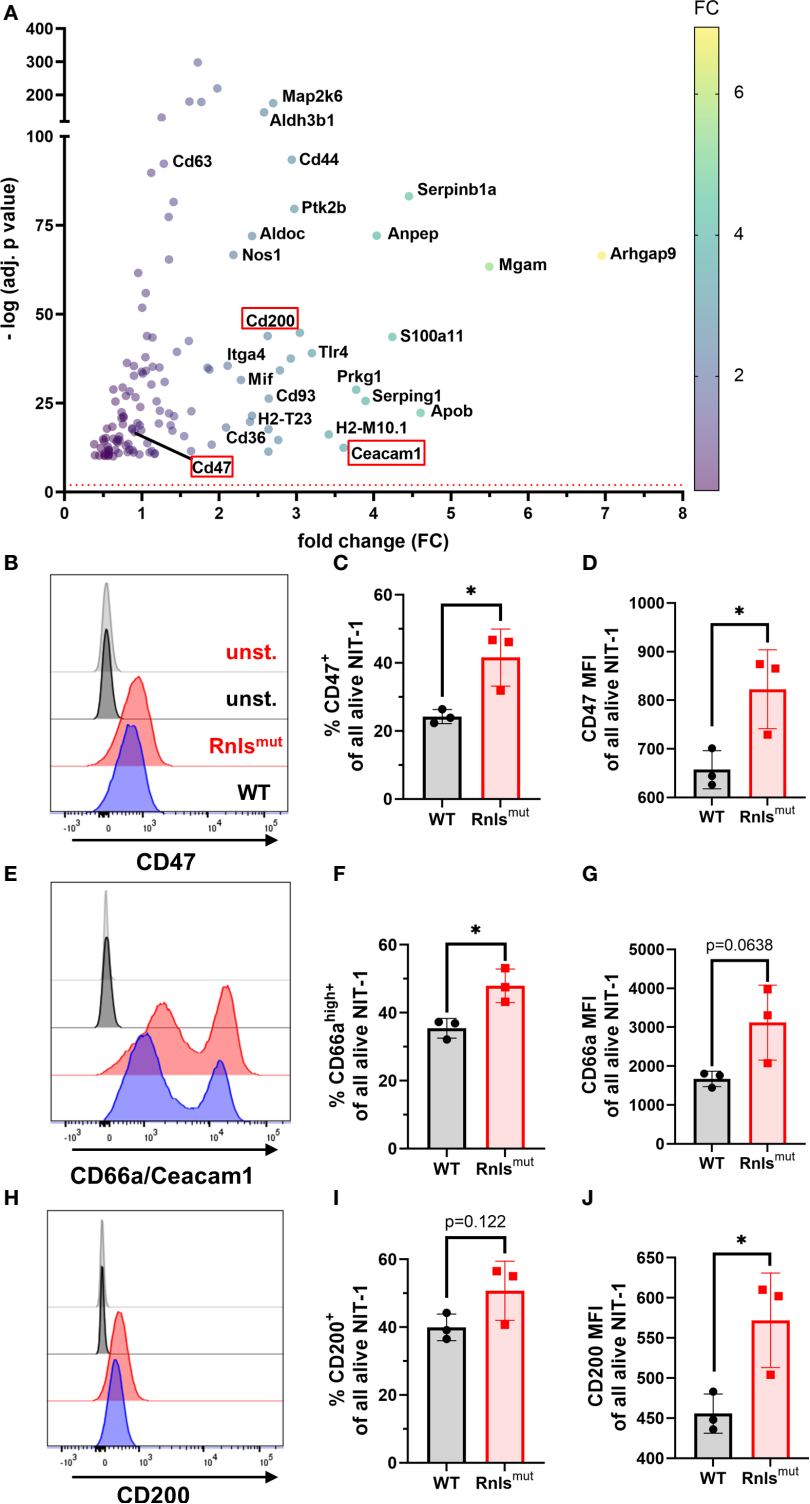
Rnls^mut^ NIT-1 beta cells show upregulated expression of key NK inhibitory ligands on their cell surface **(A)** RNA sequencing data showing the top 1,500 upregulated immune cell function-related genes (defined in GSEA-MSigDB) in Rnls^mut^ NIT-1 beta cells in comparison to WT control. Cell surface molecules known to reduce NK cell activity are highlighted in a red box. The red dotted line indicates the threshold for significantly changed genes [−log10 (*p* adj.) ≥ 2]. The fold change of gene expression is represented by colored dots from low (purple) to high (yellow). Raw data used in this panel were obtained from a bulk RNA sequencing experiment performed previously (10). **(B–J)** Representative histogram plots or quantifications of indicated inhibitory NK cell ligands expressed on the cell surface of Rnls^mut^ and WT NIT-1 beta cells characterized by flow cytometry. Results represent the mean ± SD from one out of three independent experiments (*n* = 3). *p < 0.05 (unpaired, two-tailed *t*-test).

We demonstrated that Rnls^mut^ NIT-1 beta cells are potent modulators of NK cell activation (**Figures 1**, **2**). To explore how *Rnls*-deficient beta cells might influence the activity of NK cells, we re-analyzed bulk RNA sequencing comparing differential gene expression data of WT and Rnls^mut^ NIT-1 cells (10). Rnls^mut^ NIT-1 beta cells showed an enrichment of genes involved in immune-regulatory interactions between a lymphoid and a non-lymphoid cell (**Supplementary Figure 1J**), indicating that upregulated expression of inhibitory cell surface molecules may regulate cell contact-dependent NK cell stimulation. Next, we selected all candidates from the top 1,500 most significant upregulated genes in Rnls^mut^ NIT-1 cells that are known to be involved in modulation of immune cell activity according to the GSEA-MSigDB data base (https://www.gsea-msigdb.org). In addition to the elevated expression of CD44 and Itga4 important for cell**–**cell adhesion processes (17, 18), Rnls^mut^ NIT-1 beta cells also upregulate key immune checkpoint surface molecules such as Ceacam1 (also known as CD66a), CD47 (also known as integrin associated protein), and CD200 (also known as OX-2), which are all eminent potent inhibitors of NK cell activation (**Figure 3A**) ^19-22^. To validate if elevated mRNA expression correspond to higher protein expression, we performed cell surface staining for these three key inhibitory NK cell ligands analyzed by flow cytometry. Rnls^mut^ NIT-1 beta cells showed significant upregulated surface expression of CD47 for both percentage of cells and mean fluorescence intensity (MFI, **Figures 3B–D**). The surface expression of Ceacam1 was also significantly upregulated in terms of Ceacam1^high+^-expressing cells, whereas Ceacam1 MFI showed a tendency of elevated expression on Rnls^mut^ NIT-1 beta cells (**Figures 3E–G**). Although the percentage of cells positive for cell surface CD200 was not dramatically changed between WT and Rnls^mut^, the expression level of CD200 (MFI) on Rnls^mut^ NIT-1 beta cells was significantly upregulated (**Figures 3H–J**). These observations indicate that Rnls^mut^ NIT-1 beta cells could shape NK cell activation at least partially by the upregulation of multiple inhibitory NK cell ligands on their cell surface.

In a first attempt to investigate why Rnls^mut^ cells upregulate the expression of NK inhibitory molecules, we wondered if the changes in Rnls^mut^ beta cell metabolism towards increased glucose metabolism (10) could influence the cell surface expression of CD47. As CD47 expression has been described to be similarly regulated like the immune checkpoint PD-L1 (19), and PD-L1 is positively regulated by glycolysis (20, 21), we treated NIT-1 cells with 2-deoxy-d-glucose (2DG) to inhibit glucose metabolism. Strikingly, 2DG treatment for 48 h dramatically downregulated the surface expression of CD47 on both alive WT and Rnls^mut^ NIT-1 cells (**Supplementary Figures 3A–D**). Whereas treatment with low concentration of 2DG (1 mM) decreased the percentage of CD47-expressing Rnls^mut^ cells to about the level of untreated WT cells, small amounts of 2DG had no effect on the percentage of CD47 expression on WT cells (**Supplementary Figures 3A, C**). This observation indicates that enhanced glucose metabolism in Rnls^mut^ beta cells might be responsible for the upregulation of CD47 surface expression.

### Human RNLS^mut^ SC-derived beta-like cells recapitulate key features of NK cell regulation

**Figure 4:**
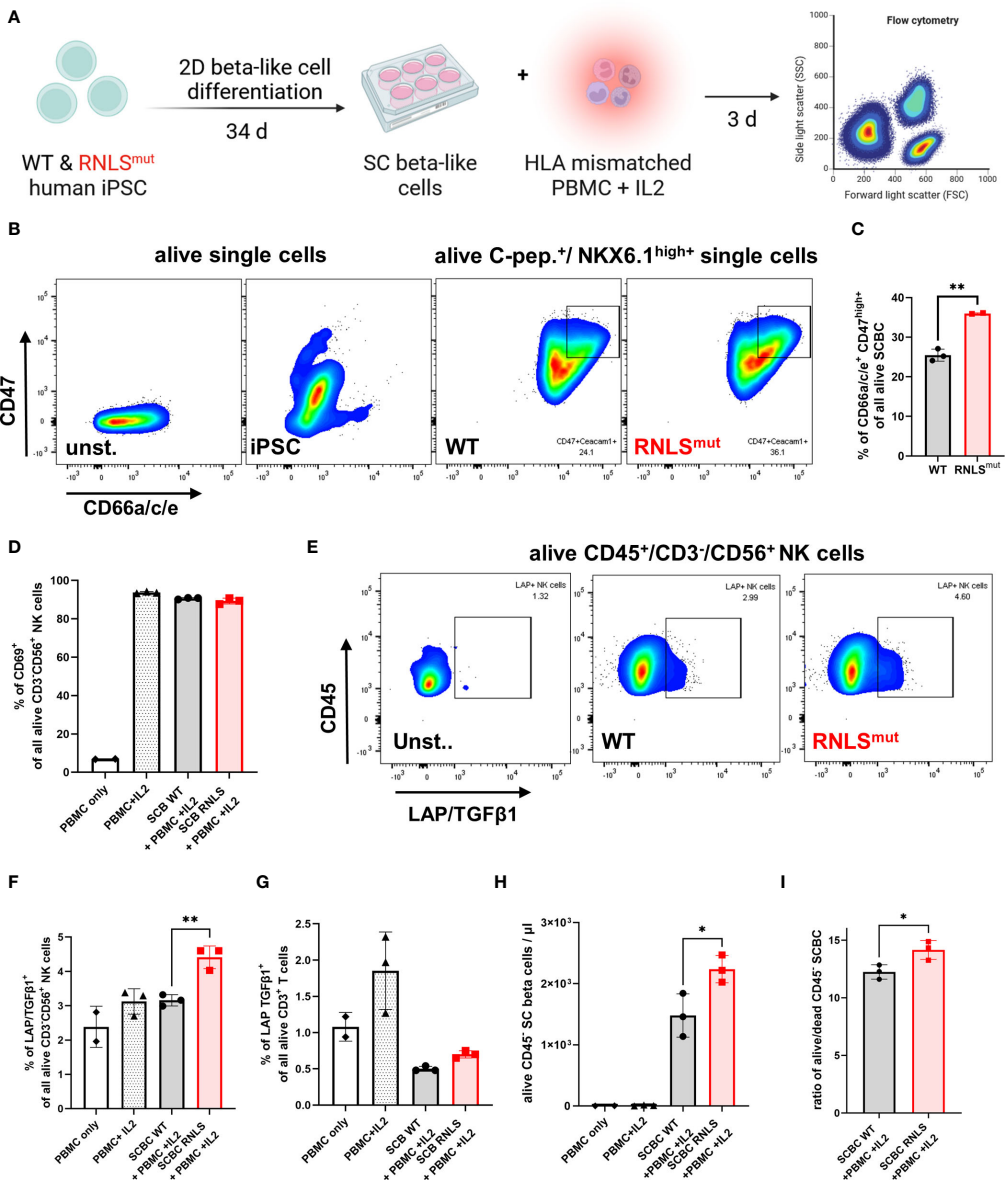
Rnls^mut^ stem cell (SC)-derived beta-like cells (SCBC) recapitulate key immune-regulatory features to modulate NK cell activity. **(A)** Schematic representation of the experimental design for SCBC differentiation and for human leukocyte antigen (HLA) mismatched PBMC co-culture approach comparing WT and RNLS-deficient SCBC. **(B, C)** Representative flow cytometry plots or quantification of indicated NK inhibitory ligands on the cell surface of C-peptide (C-pep.)^+^ NKX6.1^high+^ WT and RNLS^mut^ SCBC. **(D)** Quantification of activation marker CD69 on NK cells following 72-h incubation with IL2-stimulated HLA mismatched PBMC in co-culture with WT or RNLS^mut^ SCBC. **(E–G)** Representative flow cytometry plots or quantification of latency-associated peptide (LAP)/TGFβ1 surface expression on indicated immune cell types following IL2-stimulated HLA mismatched PBMC co-culture for 72 h. **(H, I)** Quantification of total numbers of indicated alive CD45^-^ SCBC **(H)** or alive/dead ratio of indicated SCBC **(I)** following IL2-stimulated HLA mismatched PBMC co-culture for 72 h. Results represent the mean ± SD from one out of two independent experiments (*n* = 2–3). **(C, I)** **p < 0.01(unpaired two-tailed *t*-test). (D–H)*p < 0.05, **p < 0.01 (one-way ANOVA with Dunnett *post hoc* test).

Rnls^mut^ NIT-1 beta cells strongly modulate NK cell activity towards a regulatory phenotype. To make sure that our observations are not restricted to mouse-derived NIT-1 beta cells, we also analyzed human iPSC-derived beta-like cells (SCBCs) lacking the *RNLS* gene (9) for NK regulatory characteristics (**Figure 4A**). Intracellular staining for beta cell marker demonstrated that SCs differentiate into beta-like cells regardless of genotype, as previously described (**Supplementary Figures 4A, B**) (9). Rnls^mut^ NIT-1 beta cells showed elevated surface expression of NK inhibitory molecules CD47, Ceacam1, and CD200 (**Figure 3**). In line with Rnls^mut^ NIT-1 beta cells, RNLS^mut^ SCBCs also demonstrated significantly upregulated expression of NK inhibitory ligands CD47 and CD66a/c/e, whereas, in contrast to NIT-1 beta cells, CD200 surface expression was not elevated on RNLS^mut^ SCBCs (**Figures 4B, C**, **Supplementary Figures 4C–H**). We co-cultured WT and RNLS^mut^ with allogenic peripheral blood mononuclear cells (PBMCs) stimulated with IL2 for 3 days (**Figure 4A**). Although surface expression of the NK cell activation marker was only modestly changed in this highly stimulatory allogenic setting (**Figure 4D** and data not shown), RNLS^mut^ SCBCs significantly upregulated the immune-regulatory cytokine TGFβ1 on human CD3^-^CD56^+^ NK cells (**Figures 4E, F**). These findings clearly show that human beta cells lacking *RNLS* acquire key characteristics to induce a regulatory NK cell phenotype. Because we previously demonstrated that Tgfβ1 expression is elevated on other NOD-derived immune cells such as CD3^+^ CD4^+^ T cells when infiltrating in Rnls^mut^ beta cell grafts (10), we stained human CD3^+^ T cells for membrane-bound TGFβ1 as well. Indeed, PBMC-derived CD3^+^ T cells showed elevated surface expression of LAP/TGFβ1 when co-cultured with RNLS^mut^ SCBCs compared to WT SCBCs (**Figure 4G**). Finally, we tested if RNLS^mut^ SCBCs had higher resistance to immune-mediated destruction by IL2-stimulated allogenic PBMCs. The total number of alive CD45 negative SCBCs as well as the ratio of alive/dead SCBCs were significantly higher for RNLS^mut^ SCBC compared to WT indicating that the protective immune-regulatory effects of *RNLS*-deficient beta cells also translate, at least to some extent, to the human system (**Figures 4H, I**). Our results highlight that knocking out a single gene in beta cells can drive multifaceted modulation on the immunogenicity of beta cells to prevent autoimmunity.

## Discussion

In this work, we provide compelling evidence supporting the critical involvement of NK cells in orchestrating the immune-regulatory milieu surrounding Rnls^mut^ beta cells. In our previous study, we already observed a marked reduction in NK cell frequency within Rnls^mut^ beta cell grafts compared to WT counterparts, suggesting a potential link between NK cells and the protective effects conferred by Rnls^mut^ beta cells (10). Here, further characterization of graft-infiltrating NK cells revealed distinct gene expression profiles, with NK cells derived from Rnls^mut^ beta cell grafts exhibiting upregulated expression of immune-regulatory Tgfβ1, reinforcing their role in mitigating autoimmune responses. Dysregulation of Tgfβ1 signaling has been implicated in various autoimmune disorders, highlighting its crucial role in maintaining immune homeostasis and preventing autoimmunity (22). Notably, depletion of NKp46^+^ cells abrogated the protective effects of Rnls^mut^ beta cells against autoimmunity, underscoring the indispensability of innate lymphoid cells, especially NK cells, in mediating immune regulation within the beta cell graft microenvironment.

Our previous work has established that PD-L1 blockade abrogates the survival advantage of Rnls^mut^ beta cells, implicating the PD-1/PD-L1 checkpoint as a crucial mediator of immune regulation in beta cell graft survival. In extension of our previous finding, our current observations elucidate the role of NKp46^+^ innate lymphoid cells in shaping the phenotype of APCs within the beta cell grafts. We observed a significant reduction in the frequency of mature MHCII^high+^ APCs within Rnls^mut^ beta cell grafts, which was completely dependent on the presence of graft-infiltrating NKp46^+^ immune cells. Enhanced secretion of Tgfβ1 derived from NK cells most likely induces the tolerogenic APC phenotype in Rnls^mut^ beta cell grafts. It is well established that Tgfβ1 prevents dendritic cell maturation including MHCII upregulation, underscoring the intricate cross-talk between NK cells and APCs we have observed here (23). In the context of lung cancer, the interplay between NK cells and APCs has also been noted to have immune-regulatory implications, including impairments in MHCII expression and in modulation of immune checkpoint pathways (24). Of note, and in line with our observations, Tgfβ1 has been described as potent inducer of PD-L1 expression on APCs in pancreatic islet transplantation (11). This suggests that NK cells play a pivotal role in shaping the tolerogenic phenotype of APCs, thereby contributing to the overall immune-regulatory environment conducive for survival of beta cell grafts.

In this study, *in vitro* co-culture experiments provided mechanistic insights into the direct modulation of NK cell activity by Rnls^mut^ beta cells. Beta cells deficient for *Rnls* exhibited the ability to attenuate NK cell activation and cytotoxicity, while concurrently promoting the expression of anti-inflammatory Tgfβ1. Interestingly, Rnls^mut^ beta cells also upregulated inhibitory immune checkpoint molecules such as CD47 and Ceacam1, further corroborating their role in dampening NK cell activity and immune responses (25–29). Overexpression of CD47 on hypo-immunogenic MHCI/II-deficient islets have already been described to play a significant role in the inhibition of NK cells to prevent rejection of engrafted cells in humanized mice (30). However, other surface molecules or immune-modulatory alterations such as cellular metabolism in Rnls^mut^ beta cells may contribute to the inhibition of NK cell activity as well. The results obtained in this study indicate that enhanced glucose metabolism in Rnls^mut^ beta cells contributes to elevated expression of CD47, as the inhibition of glycolysis by 2DG treatment strongly reduced cell surface expression of CD47. It has been shown that PD-L1 expression is regulated by glucose metabolism (20), but it has not been described before that CD47 surface expression is regulated similarly by glucose metabolism. It is known that the expression of PD-L1 and CD47 is often regulated by similar mechanisms such as by stimulation with Ifng (19). However, future studies have to investigate the molecular mechanism how glucose metabolism is linked to the upregulation of CD47 surface expression on beta cells.

Moreover, our findings extend to human beta-like cells derived from induced pluripotent SCs, highlighting the translational relevance of our observations. RNLS^mut^ SCBCs exhibited similar patterns of NK cell regulation and upregulated expression of inhibitory immune checkpoint molecules CD47 and CD66a/c/e. In addition to mouse-derived NIT-1 cells, RNLS^mut^ SCBCs also mediate the induction of TGFβ1 on both human NK cells and T cells (10), further affirming the conservation of immune-modulatory mechanisms across species. This is in line with our previous study where we demonstrated that RNLS^mut^ SCBCs are resistant to stress-induced apoptosis similar to *Rnls*-deficient NIT-1 beta cells (9).

Overall, our study elucidates the intricate interplay between Rnls^mut^ beta cells and NKp46^+^ NK cells in orchestrating protective immune regulation, unveiling potential targets for therapeutic intervention in autoimmune diabetes. By unraveling the mechanisms underlying immune modulation within the beta cell microenvironment, our findings pave the way for the development of novel strategies aimed at preserving beta cell function and improving graft survival in T1D.

## Research design and methods

### Mice

NOD (nonobese diabetic), NOD.scid (NOD.Cg-Prkdc^scid^/J), and NSG (NOD.Cg-Prkdc^scid^ Il2rg^tm1Wjl^/SzJ) mice were purchased from The Jackson Laboratory. Animals were housed in pathogen-free facilities at the Joslin Diabetes Center and all experimental procedures were approved and performed in accordance with institutional guidelines and regulations (IACUC protocol number 2013–03).

### Cell lines and induced pluripotent stem cell-derived beta-like cell differentiation

NIT-1 cells were obtained from ATCC (cat # CRL-2055). Cells were maintained in DMEM, high glucose, and pyruvate (cat # 11–995-073; Gibco), supplemented with 2 mM L-glutamine (cat # 25–030-081; Gibco),10% FCS (cat # 10–082-147; Gibco), 50 μM 2-mercaptoethanol (cat # 60–24-2; Sigma-Aldrich), and penicillin/streptomycin (cat # 15140122; Thermo Fisher Scientific) in a 37°C incubator with 5% CO_2_. Rnls^mut^ NIT-1 cells were generated previously (10). Human induced pluripotent stem cells (iPSCs) obtained from Local donor 3 with T1D were carefully maintained and differentiated following robust protocols tailored for beta cell differentiation (31). Cultured in planar form using mTeSR medium (cat# 85850; Stem Cell Technologies), iPSCs subjected to daily media changes to sustain optimal growth conditions. Employing a 2-D differentiation protocol (32), iPSCs were seeded at stage 0 onto culture plates at a density of 0.8 × 105 cells/cm^2^, supplemented with 10 μM Y-27632 (cat # DNSK-KI-15–02; DNSK International) to enhance cell survival and adherence. Quality control measures were rigorously implemented at each differentiation stage, utilizing immunofluorescence (IF) or flow cytometry analyses with specific differentiation marker to assess differentiation efficiency and cellular identity (data not shown). For effective cell dissociation, TrypLE Express (cat # 12604013; Gibco) treatment was administered at 37°C for 10 min to disrupt cell**–**cell adhesions, facilitating the generation of single-cell suspensions. Following dissociation, cells were promptly fixed and stained as per established procedures (33) to visualize cellular marker indicative of beta cell differentiation. Quality control at stage 6 involved the use of antibodies targeting C-peptide and NKX6.1 analyzed by flow cytometry (see **Table 1**). Ethical approval for all human cell experiments was meticulously obtained from the Harvard University Institutional Review Board (IRB) and the Embryonic Stem Cell Research Oversight (ESCRO) committees, underscoring our commitment to ethical research practices and compliance with regulatory guidelines. RNLS^mut^ iPSCs from an individual with T1D (Local donor 3) were obtained, generated, and gifted by the Melton Lab (Harvard University) as described before (9).

**Table 1:** Antibodies used for flow cytometry.

Antibody specificity	Reactivity	Clone	Cat #	Vendor
C-peptide Alexa Fluor 647	Human	U8–424	565831	BD Biosciences
CD3 Brilliant Violet 785	Mouse	17A2	100232	BioLegend
CD3 Brilliant Violet 605	Mouse	17A2	100237	BioLegend
CD3 PE-Cy5	Human	HIT3a	300310	BioLegend
CD4 APC	Mouse	RM4–5	100516	BioLegend
CD8 FITC	Mouse	53–6.7	100706	BioLegend
CD11b APC-Cy7	Mouse	M1/70	101226	BioLegend
CD11c Brilliant Violet 711	Mouse	N418	117349	BioLegend
CD19 Pacific Blue	Mouse	1D3/CD19	152416	BioLegend
CD44 APC-Cy7	Mouse	IM7	103028	BioLegend
CD44 PE	Human	C44Mab-5	397504	BioLegend
CD45 PE-Cy7	Mouse	30-F11	103114	BioLegend
CD45 PE-Cy7	Human	2D1	368532	BioLegend
CD47 APC	Mouse	Miap301	127514	BioLegend
CD47 PE-Cy7	Human	CC261	323113	BioLegend
CD56 Brilliant Violet 605	Human	HCD56	318334	BioLegend
CD66a	Mouse	Mab-CC1	134540	BioLegend
CD66a/c/e Brilliant Violet 421	Human	ASL-32	342313	BioLegend
CD69 PE-Cy5	Mouse	H1.2F3	104510	BioLegend
CD69 Brilliant Violet 421	Human	FN50	310930	BioLegend
CD107a FITC	Mouse	1D4B	121606	BioLegend
CD200 (OX2) APC	Mouse	OX90	123810	BioLegend
CD200 (OX2) Brilliant Violet 711	Human	OX-104	329222	BioLegend
F4/80 FITC	Mouse	BM8	123108	BioLegend
I-A^k^ (MHC class II) PE	Mouse	10–3.6	109908	BioLegend
LAP/TGF-β1 PE	Mouse	TW7–16B4	141404	BioLegend
LAP/TGF-β1 FITC	Human	S20006A	300010	BioLegend
Ly-6C Brilliant Violet 421	Mouse	HK1.4	128032	BioLegend
Ly-6G Brilliant Violet 785	Mouse	1A8	127645	BioLegend
NKp46 Brilliant Violet 711	Mouse	9E2	331936	BioLegend
NKp46 PerCP/Cyanine5.5	Mouse	29A1.4	137610	BioLegend
NKX6.1 PE	Human	R11–560	563023	BD Biosciences
PD-L1 PerCP/Cyanine5.5	Mouse	10F.9G2	124334	BioLegend

### Bulk RNA sequencing

Bulk RNAseq of WT and Rnls^mut^ NIT-1 cells were performed previously (10). In brief, triplicates of 2 × 106 NIT-1 cells were used for RNA isolation using Zymo Quick-RNA miniprep plus kit (cat # R1058; Zymo Research), following the manufacturer’s protocol. RNA libraries were prepared, and subsequently quality control tested by Novogen Corporation Inc. Sequencing was performed on an Illumina NovaSeq 6000 sequencing system for a >20 million read data output (Novogen Corporation Inc.). For enrichment analysis of immune system-related genes (according to GSEA-MSigDB, https://www.gsea-msigdb.org) the 1,500 most significant upregulated genes in *Rnls* mutant *vs.* WT control are shown (**Figure 3A**).

### NK cell/NIT-1 beta cell co-culture

NK cells were obtained from spleens of 8- to 16-week-old non-diabetic NOD/ShiLtJ mice purchased from The Jackson Laboratory (strain #: 001976; three to four spleens were used per experiment to collect sufficient amount of NK cells) using the NKp46^+^ NK Isolation Kit (cat # 130–115-818; Miltenyi Biotec). For co-culture experiments, 10^4^ NIT-1 cells were seeded in each well of a 96-well flat bottom (trans-well assays) or round-bottom (co-culture assays) plate in 100 μl of NK cell medium (DMEM, high glucose, pyruvate (cat # 11–995-073; Gibco), supplemented with 10% FCS (cat # 10–082-147; Gibco), 2 mM L-glutamine (cat # 25–030-081; Gibco), and penicillin/streptomycin (cat # 15140122; Thermo Fisher Scientific) following incubation for 3 days in a 37°C incubator with 5% CO_2_. In trans-well assays, NK cells were fluorescently labeled using *Tag-it Violet* proliferation dye according to the manufacturers’ protocol (cat # 425101; BioLegend) and 5 × 10^4^ NK cells were added to NIT-1 cell-containing 96-well plates either in direct co-culture or on a trans-well insert (cat # 3380; Corning). Recombinant mouse (m)IL-2 (cat # 575406; BioLegend) was added to stimulate NK cells with a total of 20–100 ng/mL final concentration as indicated. For trans-well assays, 100 ng/mL mIL2 was freshly added daily to achieve stronger NK cell proliferation. To investigate NK cell proliferation, NK cells were stained with Tag-it Viole Proliferation and Cell Tracking Dye (cat # 425101; BioLegend) according to the manufacturer’s instructions. Conditioned media experiments were performed by the addition of medium derived from WT or Rnls^mut^ NIT-1 cells with a ratio of 1:1 (conditioned media:NK cell media). Additional conditioned medium was supplemented following 24 h (final ratio 3:1) and 48 h (final ratio 4:1) each time in combination with repetitive IL2 stimulation (100 ng/mL). Supernatants were collected following centrifugation for 5 min at 300 g and stored at −20°C. ELISA for Ifng (cat # MIF00; R&D Systems) and Tgfβ1 (cat # DB100C; R&D Systems) were performed according to the manufacturer’s instructions. In all experiments, cells were co-cultured in a 37°C incubator with 5% CO_2_ for 72 h, then collected and stained as described in the “Flow cytometry” section with indicated antibodies.

### PBMC/SCBC co-culture

PBMCs collected from allogenic donors (under protocol Joslin CHS # 2013–10) in EDTA tubes were isolated using Lymphoprep density gradient medium (cat # 07801; Stem Cell Technologies). A total of 5 × 10^6^ PBMCs were transferred into each well of a 6-well plate with fully confluent WT or RNLS^mut^ SCBC in 3 mL of HEPES buffered PBMC medium [RPMI-1640 with penicillin/streptomycin, L-glutamine, and HEPES (cat # ABI-Custom American BioInnovations), supplemented with 10% human AB serum (cat # H5667; Millipore Sigma) and 100 ng/mL recombinant human IL2 (cat # 589104; BioLegend)] following incubation at 37°C with 5% CO_2_. Following co-culture for 72 h, cells were detached with Trypsin-EDTA (cat # 25–200-056; Gibco) and filtered using a 70-μm pore size strainer twice to obtain a single-cell solution. Collected cells were stained with indicated antibodies listed in **Table 1** as described in the “Flow cytometry” section. The quantification of alive CD45^-^ SCBCs was performed by using Precision Count Beads (cat # 424902; BioLegend). Cells were collected and stained as described in the “Flow cytometry” section with indicated antibodies.

### Splenocyte transfer mouse model and graft isolation

WT and Rnls^mut^ NIT-1 beta cells (10^7^ each) were injected subcutaneously (s.c.) into opposite flanks of the same immunodeficient NOD.Cg-Prkdc^scid^/J (NOD; strain #: 001303) or NOD.Cg-Prkdc^scid^ Il2rg^tm1Wjl^/SzJ (NSG; starin #: 005557) mice purchased from The Jackson Laboratory. Four days later, autoreactive splenocytes from recently diabetic NOD mice were isolated and red blood cells were lysed using 5 mL of ACK lysing buffer (cat # A1049201; Thermo Fisher Scientific) for 4 min at RT. Lysis was stopped using 5 mL of PBS/10% FCS followed by two washing steps using PBS only. Splenocytes were filtered two times in total through a strainer with 70 μm pore size to obtain a single-cell solution. For NK cell depletion, NKp46-expressing splenocytes were removed by using the anti-NKp46 MicroBead kit (cat # 130–095-390; Miltenyi Biotec) according to the manufacturer’s instructions. Splenocytes (10^7^/mouse) were injected intravenously (i.v.) into NIT-1 cell-bearing NOD.scid/NSG recipient mice to transfer autoimmune beta cell killing for a total of 17 days. NIT-1 beta cell graft isolation was performed as described before (10). In brief, grafts were isolated and scaled on an analytical balance 17 days after splenocyte transfer. Grafts were cut into small pieces and digested using HEPES buffered RMPI 1640 (cat # R4130–10L; Sigma-Aldrich) supplemented with 1 mg/mL Collagenase D (cat # 11088858001; Sigma-Aldrich), 20 μg/mL DNase I (cat # EN0521; Thermo Fisher Scientific), 2% FCS, and 50 μg/mL lipopolysaccharide neutralizing agent Polymyxin B sulfate (cat # 1405–20-5; Sigma-Aldrich) for 45 min at 37°C while shaking by maximum speed (750 rpm) on a heating block. Digested grafts were further disaggregated and filtered through a strainer with 70 μm pore size twice to obtain a single-cell solution. For flow cytometry analysis, cells were stained with the antibodies listed in **Table 1** in two separate panels (panel 1: T cells and NK cells; panel 2: myeloid cells and B cells).

### Single-cell RNA sequencing

The scRNAseq experiment was performed in our previous study (10). In brief, WT or *Rnls* mutant graft-infiltrating immune cells were FACS sorted (CD45^+^ PI^-^). Individual samples were labeled using hashtag antibodies and scRNAseq was performed on pooled samples using the Chromium Next GEM Single Cell 3’ GEM, Library & Gel Bead Kit v3.1 (cat # PN-1000213; 10x Genomics) according to the manufacturer’s instructions. Samples were super-loaded with 40,000 cells per reaction. The hashtag oligo library (HTO) was generated separately as described previously (https://citeseq.files.wordpress.com/2019/02/cell_hashing_protocol_190213.pdf). Illumina NovaSeq 6000 with approximately 1.1 billion reads was used to sequence the gene expression library, while the HTO library was sequenced separately by Illumina NextSeq with approximately 130 million reads total each. For further details on QC and analysis procedure, please refer to our previous work (10). In this study, gene set enrichment analyses of graft-infiltrating NK cells and ILC1 were performed by using the molecular signatures of hallmark gene sets provided by the MSigDB database (http://www.gsea-msigdb.org/). The indicated number of differential expressed genes within the indicated immune cell population derived from WT or Rnls^mut^ NIT-1 beta cell grafts were used as input.

### Flow cytometry

All cell surface staining procedures were performed in PBS/2 mM EDTA/10% FCS. Single-cell suspensions were pre-incubated with FcR-blocking antibody solution (cat # 156604; BioLegend) for 5 min, then stained with antibodies against indicated molecules (**Table 1**) for 20 min on ice diluted in FcR-blocking solution. Appropriate isotype controls were purchased from BioLegend. Dead cells were excluded using propidium iodide (PI, cat # R37169; Thermo Fisher Scientific) or fixable viability dye eFluor 450 (cat # 65–0863; eBiosciences). For intracellular staining (SCBC QC), the *Transcription Factor Staining Buffer Set* (cat # 00–5523-00; Thermo Fisher Scientific) was used according to the manufacturer’s instruction. In brief, following surface staining of CD47, CD66/a/c/e, and CD200 performed as described above using human FcR blocking solution (cat # 422302; BioLegend), cells were washed with PBS/2 mM EDTA/10% FCS following cell fixation with *Fixation/Permeabilization* working solution and pulse vortexing. Cells were incubated for 90 min at 4°C and washed following 2 permeabilization steps using the provided *Permeabilization Buffer*. Cells were pre-incubated in 5% human FcR-blocking antibody solution for 15 min at room temperature (RT). Cells were stained with indicated antibodies against C-peptide and NKX6.1 (working dilution of 1:20) listed in **Table 1** for 30 min at RT. Cells were washed twice, kept on ice and analyzed by flow cytometry. For the 2DG experiment to inhibit glucose metabolism in NIT-1 cells, 2 × 10^5^ cells of indicated genotypes were seeded into a 24-well plate for 48 h with or without addition of indicated concentrations of 2DG or sterile dH_2_O as control. Cell surface staining of indicated molecules were performed as described above. Prior to analysis, all cell suspensions were filtered using a 35-µm strainer (cat # 352235; Corning) to avoid cell cluster. Data were acquired on an LSRII instrument (BD Biosciences) and analyzed with *FlowJo* software v.10.6.1 (FlowJo LLC). All data are shown using log-scale axes.

### Statistical analyses

Statistical analyses were performed by unpaired or paired tests or by one-way ANOVA as indicated using GraphPad Prism v 9.0.2 software. All data are presented as the mean ± standard deviation (SD). *p* < 0.05 was considered statistically significant. Sufficient sample size was estimated without the use of a power calculation. No samples were excluded from the analyses. No randomization was used for the animal experiments. Data analysis was not blinded. All data are representative of two or more similar experiments.

## Data availability statement

The datasets presented in this study can be found in online repositories. The names of the repository/repositories and accession number(s) can be found in the article/**Supplementary Material**.

## Ethics statement

The studies involving human materials were approved by Harvard University Institutional Review Board (IRB) and the Embryonic Stem Cell Research Oversight (ESCRO) committees, and Joslin CHS # 2013-10. The studies were conducted in accordance with the local legislation and institutional requirements. The participants provided their written informed consent to participate in this study. The animal study was approved by Joslin Diabetes Center IACUC 2013-03. The study was conducted in accordance with the local legislation and institutional requirements.

## Author contributions

KB: Conceptualization, Formal Analysis, Investigation, Methodology, Supervision, Validation, Visualization, Writing – original draft, Writing – review & editing, Data curation. SW: Investigation, Methodology, Writing – review & editing, Formal Analysis. IG: Formal Analysis, Investigation, Methodology, Writing – review & editing. JL: Formal Analysis, Methodology, Writing - review & editing, Data curation. SK: Conceptualization, Funding acquisition, Supervision, Writing – review & editing. PY: Conceptualization, Funding acquisition, Project administration, Resources, Supervision, Writing – review & editing.
