# Supplementary material for: Beta cells deficient for Renalase counteract autoimmunity by shaping natural killer cell activity
Source: Front Immunol. 2024 Jun 4;15:1403752. doi: 10.3389/fimmu.2024.1403752 (PMC11225407; doi:10.3389/fimmu.2024.1403752)

Beta cells deficient for Renalase counteract autoimmunity by shaping natural killer cell activity

Kevin Bode, Siying Wei, Isabella Gruber, Jian Li, Stephan Kissler, Peng Yi

Supplemental Data Figure Legends

Supplemental Data Figure 1: $Rnls^{mut}$ graft-infiltrating NK cells and ILC1 show altered frequency and gene expression profiles. (**A** and **B**) Volcano plot showing differential gene expression (DGE) of NK cells (**A**) and type 1 innate lymphoid cells (ILC1; **B**) derived from *Rnls*-deficient *vs.* WT NIT-1 beta cell grafts. Raw data used in this figure were obtained from a 3' gene expression single cell RNA sequencing experiment performed previously [10]. Altered expression of key cytokines are marked as red dots. The gray line indicates the threshold for significant changed gene expression (-log10 (p value) ≥ 1.3). (**C**) Hallmark gene expression analysis showing the 10 most significantly changed pathways (-log10 (FDR) ≥ 2) in ILC1. For ILC1 derived from $Rnls^{mut}$ and WT grafts, 430 or 355 significantly upregulated genes were used as input, respectively. Datasets from our scRNAseq experiments are available from the Gene Expression Omnibus (https://www.ncbi.nlm.nih.gov/geo/) under accession number GSE226361 as described before. [10] (**D** and **E**) WT and $Rnls^{mut}$ NIT-1 beta cell cells were injected *s.c.* into opposite flanks of immunodeficient NOD.scid mice, followed by *i.v.* injection of autoreactive splenocytes 4 days later. Grafts were harvested prior any noticeable differences in graft sizes were observed (**E**) and immune cells were characterized by flow cytometry (**D**; See Supplemental Figure 2 for representative flow cytometry plots showing gating strategy). (**F-H**) Successful depletion of $NKp46^{+}$ cells from diabetogenic splenocytes and NIT-1 beta cell grafts. (**F**) Representative flow cytometry analysis of $CD3^{-}NKp46^{+}$ splenocytes of indicated conditions prior to *i.v.* injection into immunodeficient mice. (**G** and **H**) absolute event count of indicated graft-infiltrating immune cells. (**I**) Median fluorescence intensity (MFI) of MHC class II ($MHCII^{+}$) expressing antigen presenting cells (APC) derived from NIT-1 beta cell grafts of indicated conditions analyzed by flow cytometry. Results represent the mean of four (**A-C**), five (**G-I**; Ctrl.) or six (**D** and **E**) paired biological replicates. *** p<0.001, ** p<0.01, ns p>0.05 (paired two-tailed t-test). (**J**) RNA sequencing data showing enrichment plot for immune-regulatory interactions between a lymphoid

and a non-lymphoid cell of Rnls^{mut} NIT-1 beta cells in comparison to WT control. Raw data used in this figure were re-analyzed from a bulk RNA sequencing experiment performed previously [10].

Supplemental Data Figure 2: Gating strategy to identify indicated cell subsets of graft-infiltrating immune cells. Representative flow cytometry plots showing the overall gating strategy.

Supplemental Data Figure 3: CD47 surface expression on NIT-1 beta cells is regulated by glucose metabolism. Quantifications of CD47 expression on the cell surface of WT (**A** and **B**) and Rnls^{mut} (**C** and **D**) NIT-1 beta cells following treatment with indicated concentrations of 2-deoxy-d-glucose (2DG) characterized by flow cytometry. Results represent the mean ± SD from one out of three independent experiments (n=3). **** p<0.001, *** p<0.001, ** p<0.01, * p<0.05, ns p>0.05, (one-way ANOVA with Dunnett post hoc test).

Supplemental Data Figure 4: SCBC deficient for *Rnls* show elevated CD47 and CD66a/c/e surface expression. Representative flow cytometry plots or quantifications of indicated beta cell marker or inhibitory NK cell ligands expressed on RNLS^{mut} and WT induced pluripotent stem cell (iPSC)-derived beta-like cells (SCBC) characterized by flow cytometry. Results represent the mean ± SD from one out of two independent experiments (n=2-3). ** p<0.01, * p<0.05, ns p>0.05 (unpaired, two-tailed t-test).

Supplemental Data Figure 1

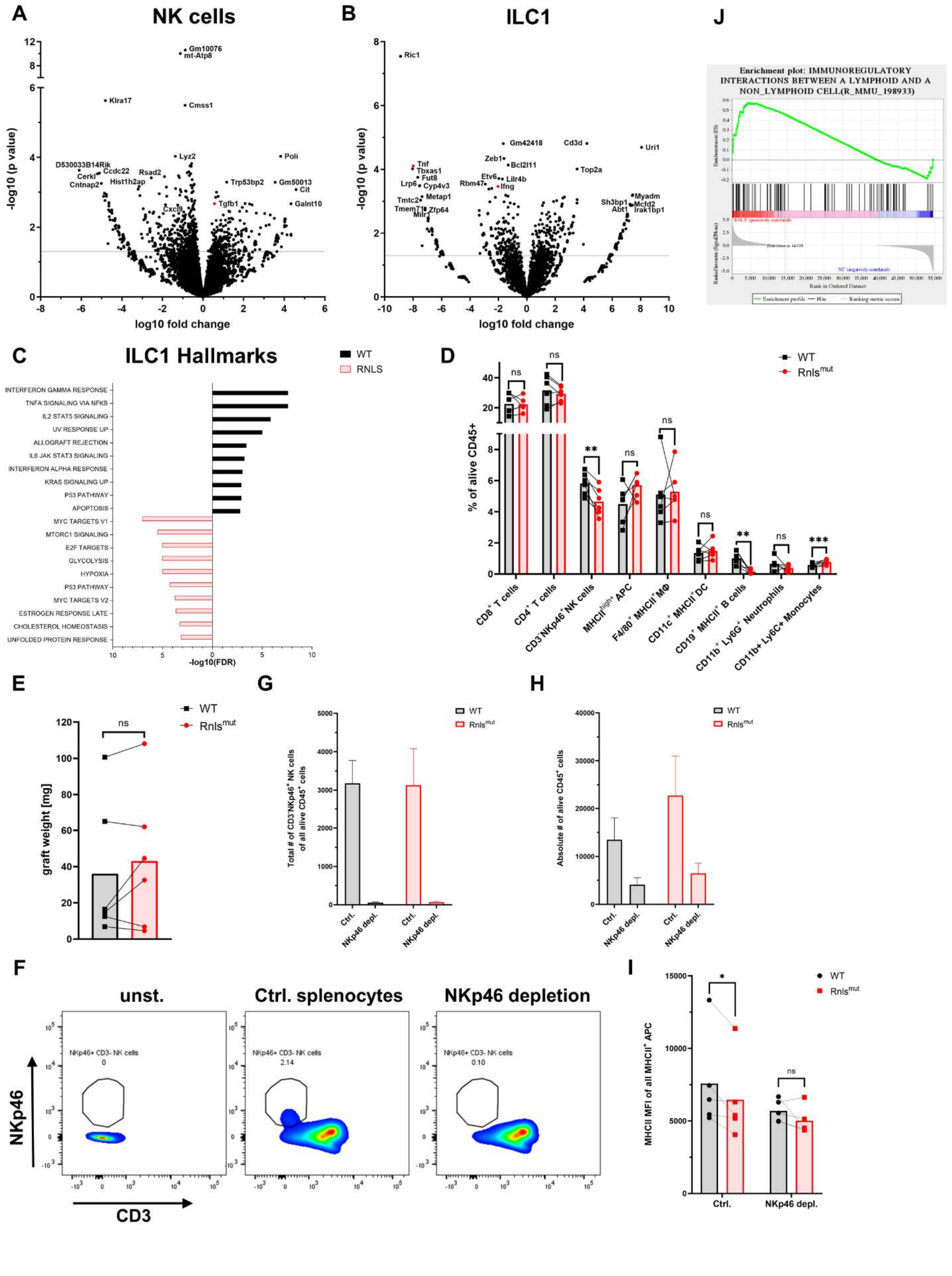

Supplemental Data Figure 2

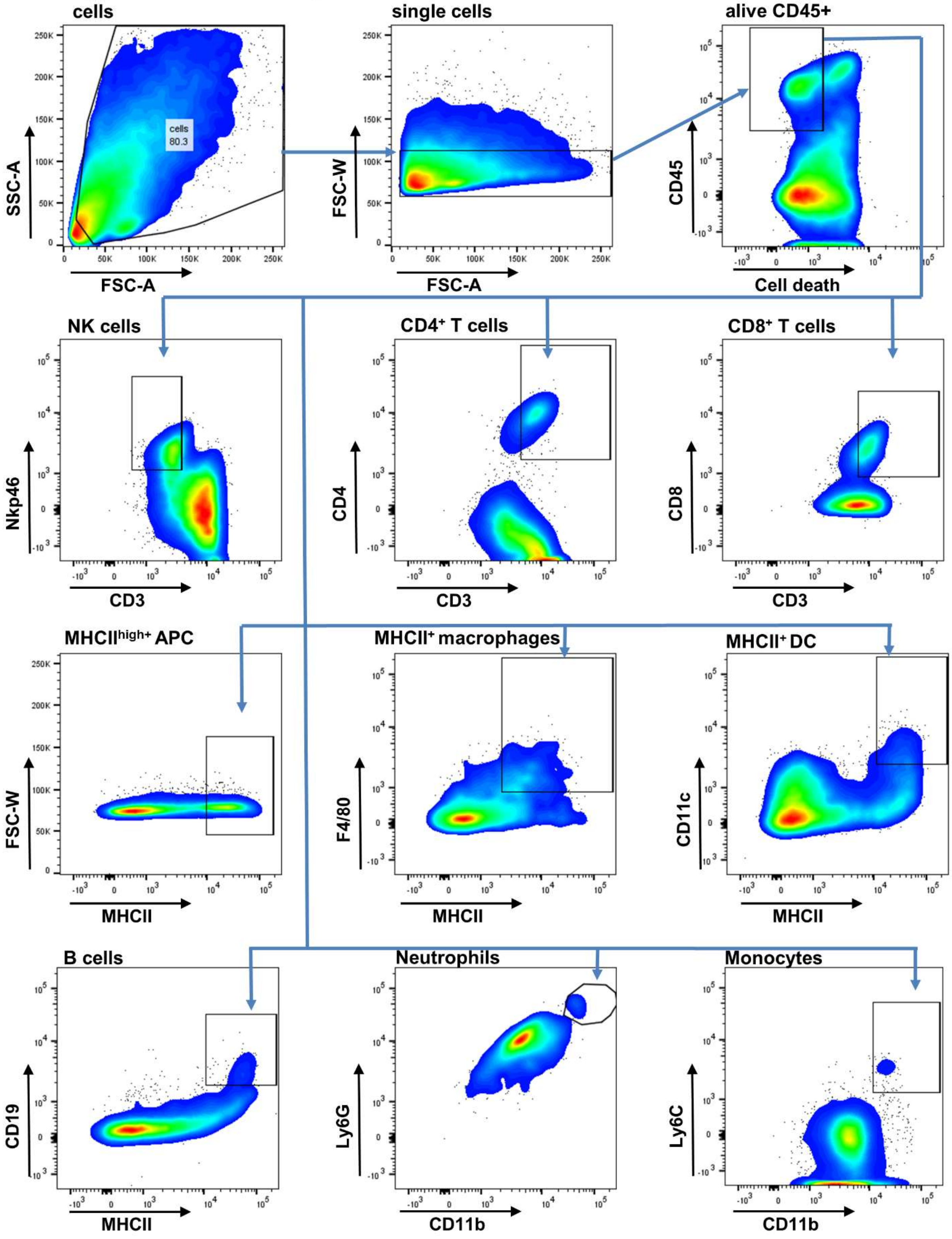

Supplemental Data Figure 3

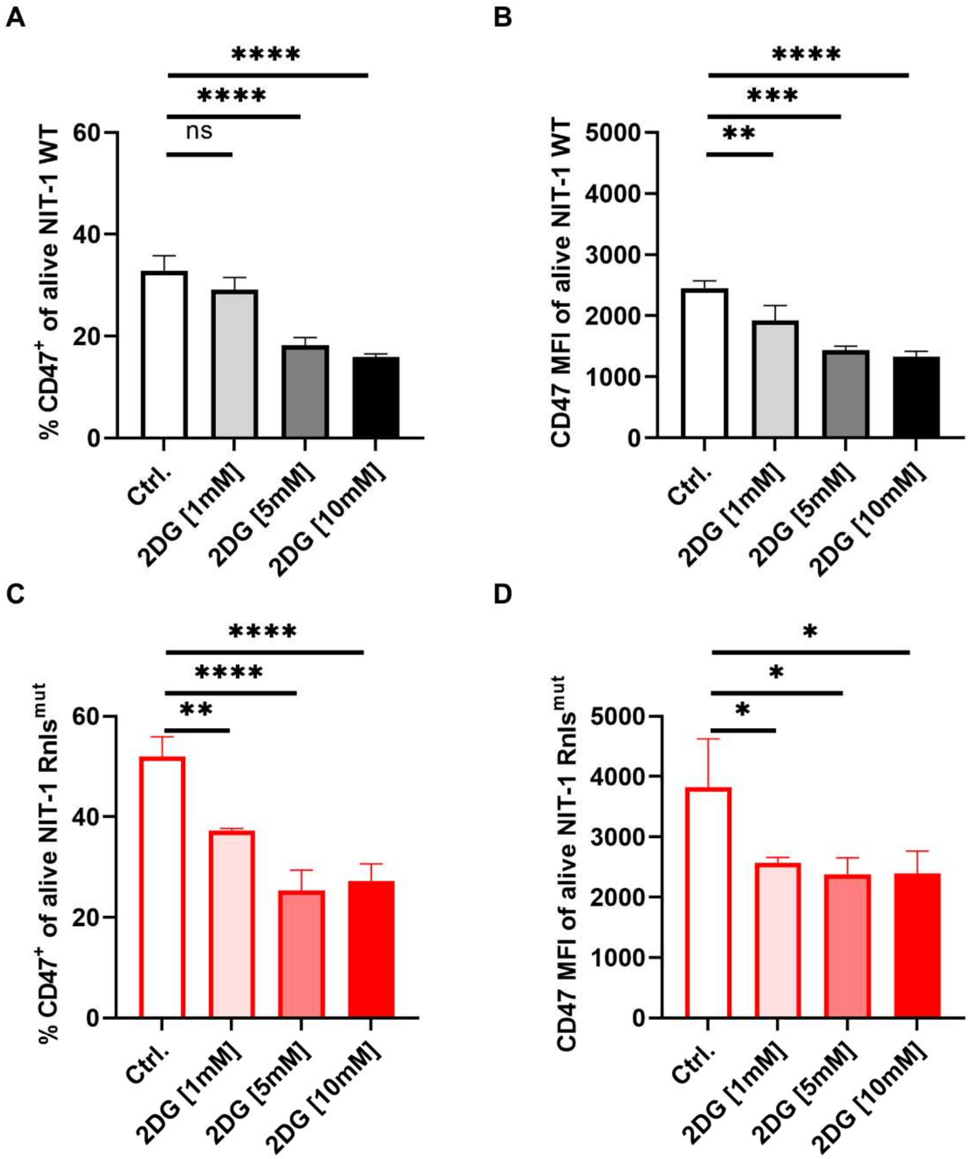

Supplemental Data Figure 4

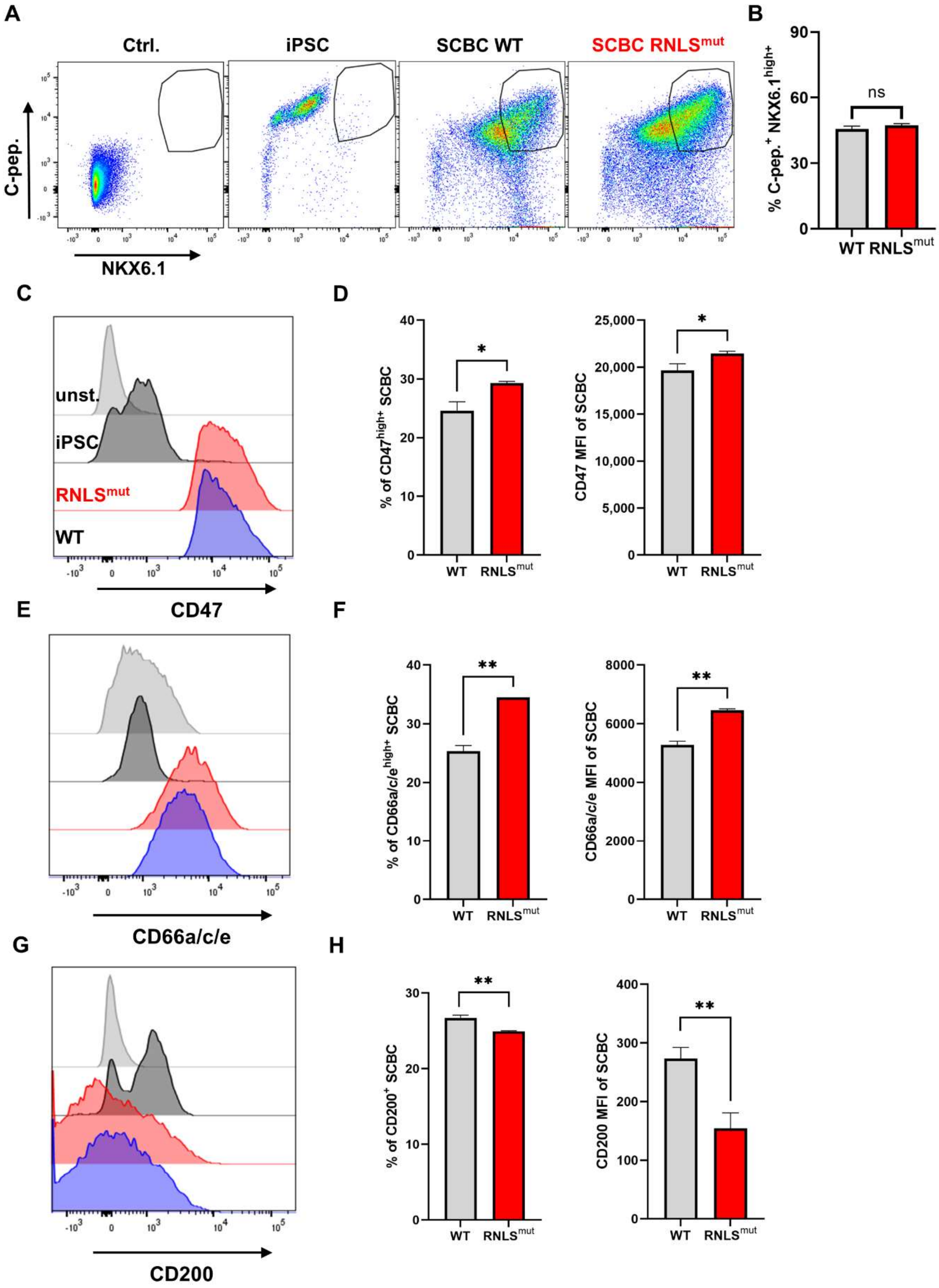

Supplement: Supplementary file 1 [file DataSheet_1.pdf]
